# Supplementary material for: Mice Microbiota Composition Changes by Inulin Feeding with a Long Fasting Period under a Two-Meals-Per-Day Schedule
Source: Nutrients. 2019 Nov 16;11(11):2802. doi: 10.3390/nu11112802 (PMC6893728; doi:10.3390/nu11112802)
Supplement: Supplementary file 1 [file nutrients-11-02802-s001.pdf]

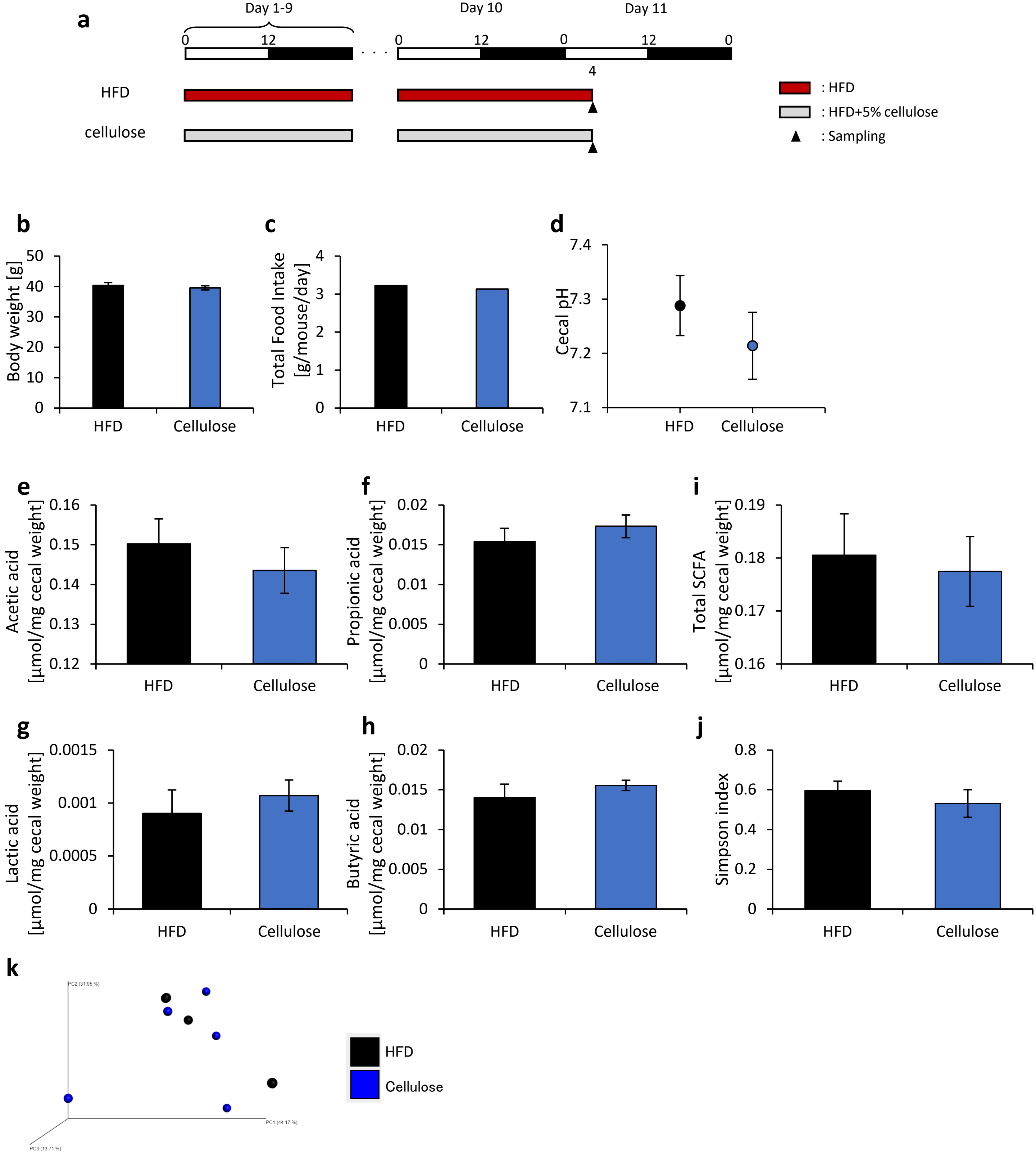

	Statistic value	p-value
HFD vs Cellulose	0.982	0.503

Figure S1

a

All data in experiment 1

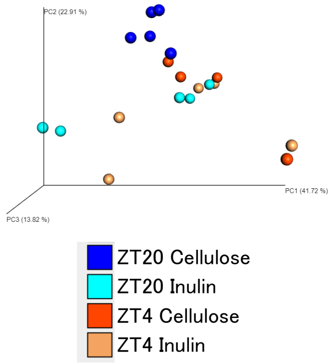

b

Effect of Food at ZT20

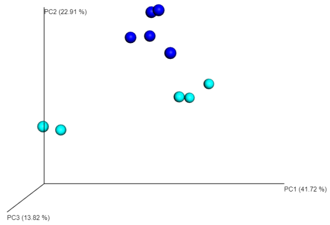

	Statistic value	p-value
Cellulose vs inulin	3.439	0.005

c

Effect of Food at ZT4

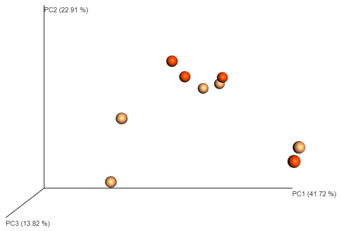

	Statistic value	p-value
Cellulose vs inulin	0.706	0.648

Figure S2

a

All data in experiment 2

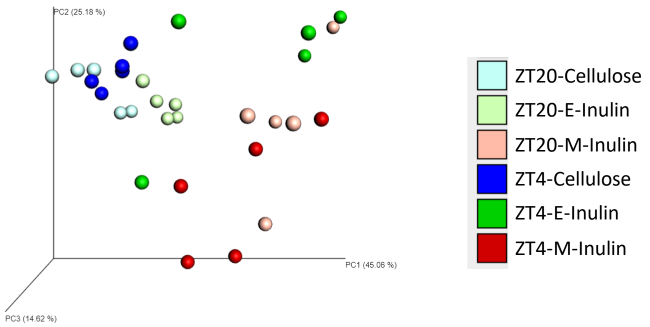

b At ZT20

Cellulose vs M-inulin

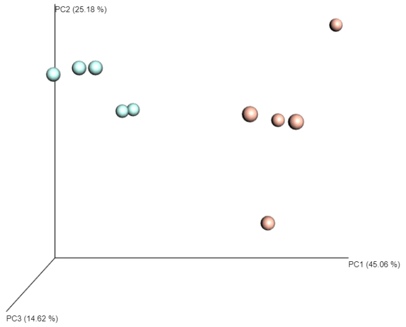

	Statistic value	p-value
Cellulose vs M-inulin	10.96	0.010

Cellulose vs E-inulin

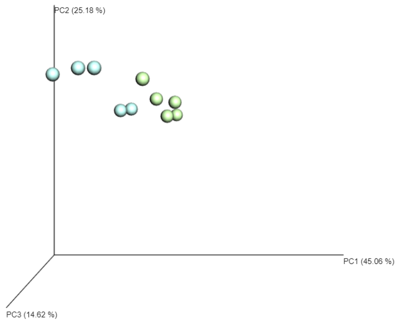

	Statistic value	p-value
Cellulose vs E-inulin	4.051	0.057

M-inulin vs E-inulin

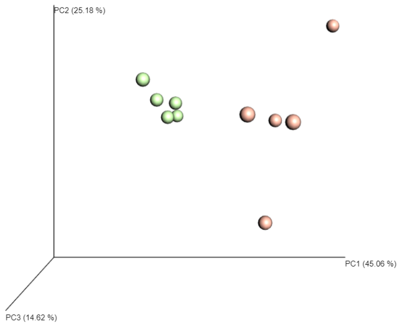

	Statistic value	p-value
M-inulin vs E-inulin	5.774	0.009

c At ZT4

Cellulose vs M-inulin

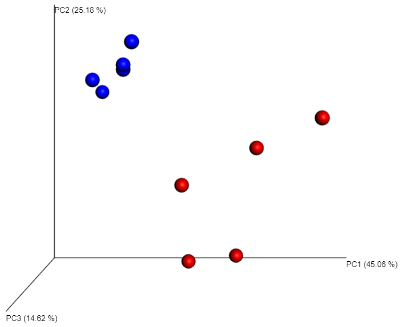

	Statistic value	p-value
Cellulose vs M-inulin	12.92	0.009

Cellulose vs E-inulin

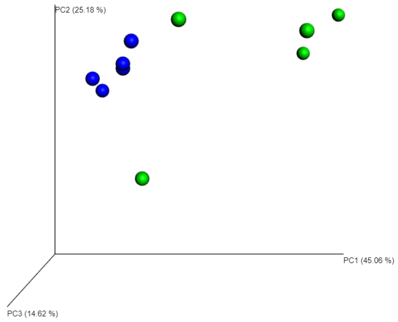

	Statistic value	p-value
Cellulose vs E-inulin	5.258	0.036

M-inulin vs E-inulin

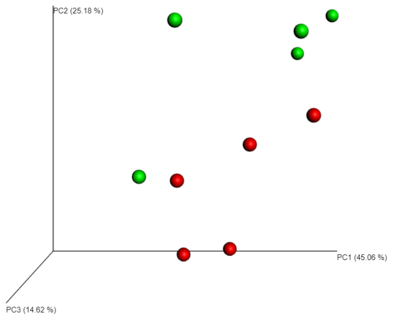

	Statistic value	p-value
M-inulin vs E-inulin	3.384	0.049

Figure S3

a

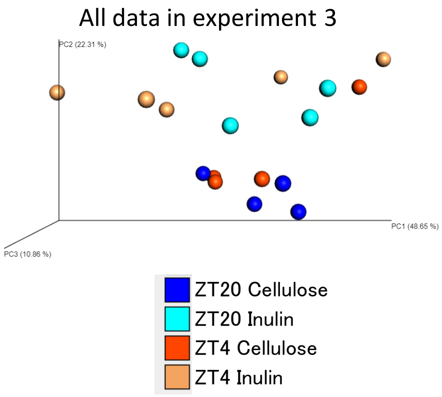

b

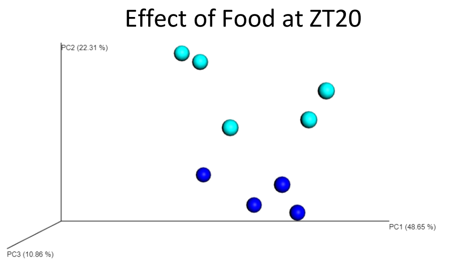

	Statistic value	p-value
Cellulose vs inulin	4.503	0.010

c

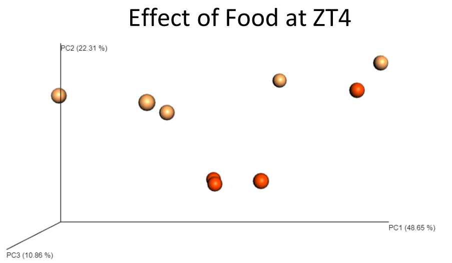

	Statistic value	p-value
Cellulose vs inulin	1.034	0.368

d

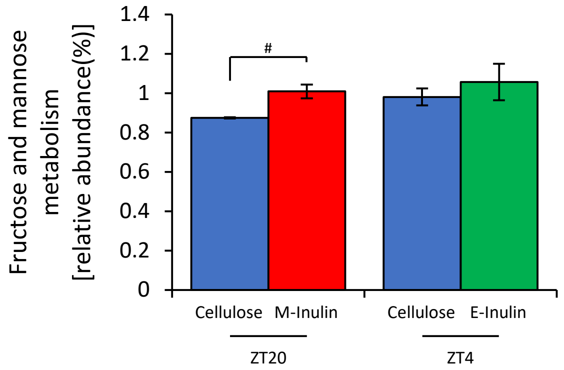

Figure S4

a

All data in experiment 4

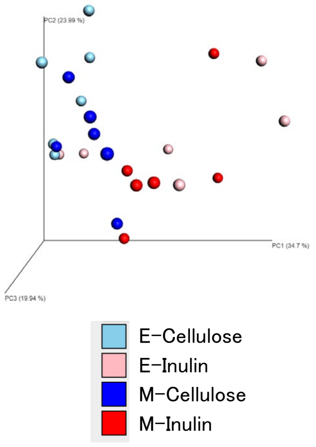

b

Effect of Food in Morning (ZT20)

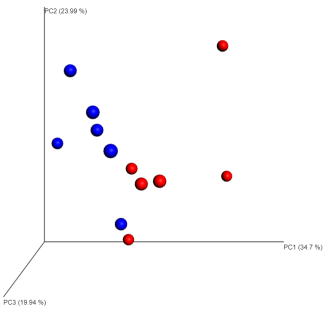

	Statistic value	p-value
Cellulose vs inulin	1.034	0.368

c

Effect of Food in Evening (ZT4)

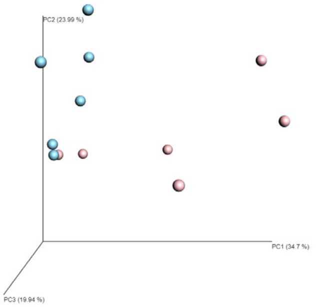

	Statistic value	p-value
Cellulose vs inulin	0.525	0.877

d

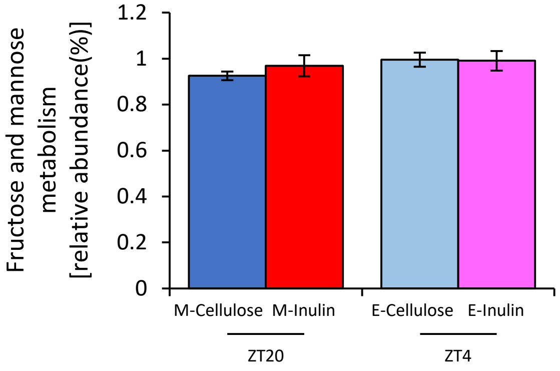

Figure S5
